# BPPV Simulation: A Powerful Tool to Understand and Optimize the Diagnostics and Treatment of all Possible Variants of BPPV

**DOI:** 10.3389/fneur.2021.632286

**Published:** 2021-03-26

**Authors:** Anita Bhandari, Herman Kingma, Rajneesh Bhandari

**Affiliations:** ^1^Vertigo and Ear Clinic, Jaipur, India; ^2^Department of Ear Nose Throat, Maastricht University, Maastricht, Netherlands; ^3^Faculty of Physics, Tomsk State National Research University, Tomsk, Russia; ^4^Department of Ear Nose Throat, Aalborg University, Aalborg, Denmark; ^5^NeuroEquilibrium Diagnostics, Jaipur, India

**Keywords:** simulation, 3D, BPPV, maneuver, semont, yacovino, otolith, rotation angle

## Abstract

BPPV is a mechanical disorder caused by the displacement of otolith debris into the semicircular canals. The treatment involves different repositioning maneuvers to bring the debris back into the utricle. This study aims to show how dynamic simulation models based on fluid dynamics and MRI, can help to visualize and understand the movement of the debris within the canals during head movement in 3D as a function of time. The user can define the rotation angle and plane at each step of the maneuver and then the model visualizes the canal and the otoconial movement in 3D. The simulation developed also allows alteration of various parameters like the rotational head acceleration, the duration of each step of the maneuver, the initial position of the otoconial debris in the canal, the size and the number of the particles and fluid dynamics of endolymph. The clod movement is visualized in such a way that it allows a better understanding of the impact and efficacy of various liberation maneuvers and why certain maneuvers might fail when not applied properly in the clinic. The model allows simulation of multi-canal BPPV. In this paper we demonstrate the power of the model applied on the maneuvers of Semont and Yacovino when executed in different ways. The model aims to provide a visual explanation for the need of specific maneuvers for each type of BPPV. The simulator presented here can be used to test the efficacy of existing maneuvers and help in the development of new maneuvers to treat different BPPV variants.

## Introduction

Benign Paroxysmal Positional Vertigo (BPPV) is amongst the most common causes of vertigo. It is a mechanical disorder of the inner ear caused by the displacement of calcium carbonate particles from the utricle into the semicircular canals. The precise mechanism and cause behind the detachment of otoconia form the utricular membrane and the migration into the canals is still unknown (1, 2).

Normally the canals are only sensitive to angular acceleration and do not sense linear accelerations because the specific mass of the cupula and endolymphe are virtual the same, close to 1.0. In BPPV patients, the presence in the canal of heavy otolith debris with a specific mass close to 2.7 makes the canal sensitive to the head orientation relative to the gravity vector. A change of head position relative to the gravity vector therefore leads to a movement of free floating heavy debris (canalolithiasis) and induces an endolymphatic flow and an associated cupula deflection leading to nystagmus. A change of head position relative to the gravity vector also induces a cupula deflection when the debris is attached to the cupula (cupulolithiasis). A patient with BPPV, either Canalolithiasis or cupulolithiasis, will, therefore, experience rotatory vertigo by head tilts because the cupula deflection is now interpreted by the brain as an angular acceleration (1–7).

Stimulation of the canals by the movement of the otoliths in it, free-floating in the canals or attached to the cupula, generates a specific eye movement called nystagmus. The direction of the nystagmus is aligned with the orientation of the canals affected. Each type of BPPV is diagnosed by observing the patterns of nystagmus induced during positioning maneuvers that have been designed to move only the involved canal in the direction of maximal gravity (2). This nystagmus direction allows us to identify which canal is affected and where the debris is located in the canal. The different otolith positions in the canals generate different characteristic nystagmus patterns.

The treatment of BPPV is based on the detection of these characteristic nystagmus patterns to decide the appropriate maneuver required to reposition the otolith debris back into the utricle. The precise debris movements in the canals have been studied and clarified by physics using various models based on the fluid dynamics of BPPV (3–9). These studies form the basis for our current understanding of the latency, direction, reversal, and fatiguability of the nystagmus as a function of time, the size and number of otoconial particles.

Repositioning procedures for BPPV depend primarily on gravity and inertia. For a successful repositioning maneuver, correct orientation, and angulation of the semicircular canals during the maneuver play a crucial role. During construction of explanations of different BPPV nystagmus patterns, movements of moving urticular otoconial debris in the three-dimensional structure of the vestibular labyrinth should be considered (10).

**Figure 1:**
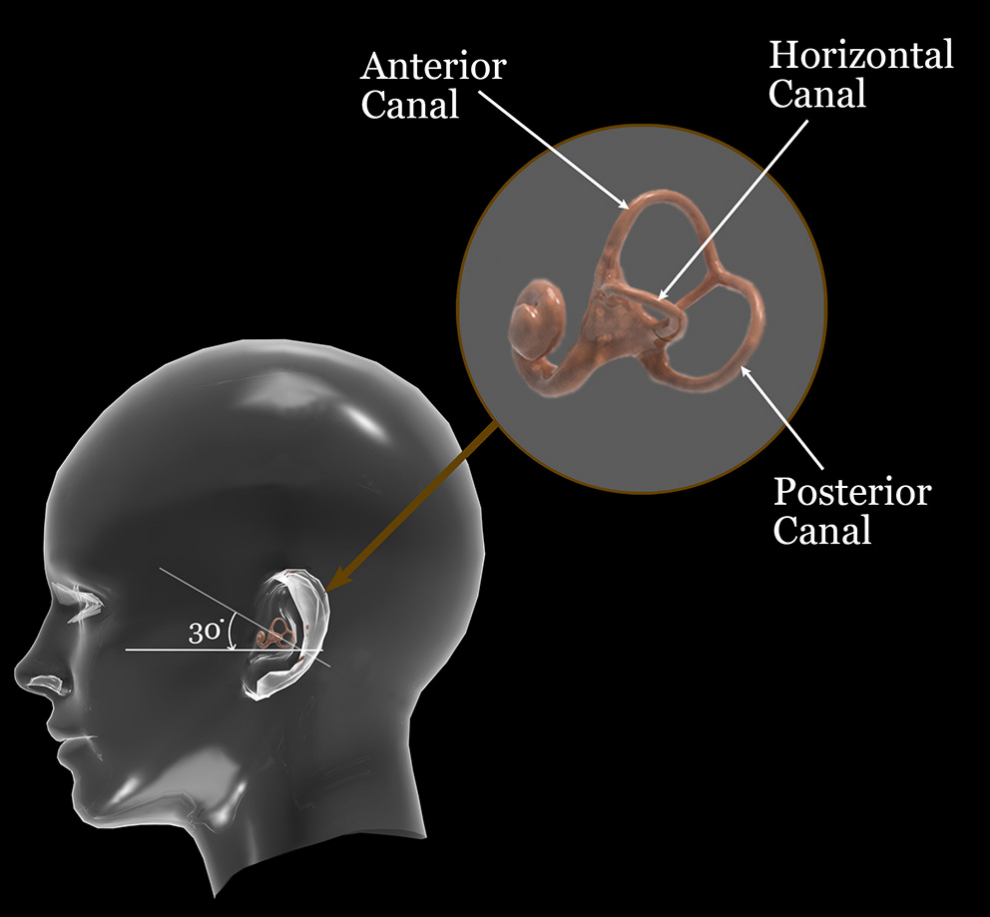
Orientation of the three semicircular canals.

Our endeavor in this article is to present a simulation to visualize the movement of the head, labyrinth, and otoconial debris in the 3-dimensional space for practical clinical use (Figure 1). It simulates the movement of the otoconial particles in the canals as a function of time and angulation during diagnostic and liberation maneuvers. In our opinion, these simulations make it possible not only to better understand but also to optimize the various diagnostic and liberation maneuvers.

Basically, two models are required to design a BPPV simulation: (1) the physics behind the debris movements and (2) the simulation algorithm based on these debris movements as a function of canal orientation relative to the gravity vector to visualize the clod movement in 3D as a function of time. For a detailed description of the physics model (1) we refer to for example the publications by Bosseli and many others (3–6). The limitations of practical application of these models lay in the limitation of exact and individual data of parameters *in vivo* involved. In reality, much is still unknown about BPPV, what is the size and variation in size of the clods, how are they distributed within a canal, are they more or less attached to the membranous labyrinth or cupula? It is very likely that these issues will vary among patients and by that limits an exact application of the models (fluid dynamics) at current. Nevertheless, the physics models based on the fluid dynamics of BPPV (3–9) form the basis for our current understanding of the latency, direction, reversal, and fatiguability of the nystagmus as a function of time. The ideal would be to optimally visualize the otolith movement in any individual patient real time. However, at present, it is still not possible to visualize the debris by MRI or CT and there are as said too many unknown variables for extrapolation of the fundamental models to the clinical reality.

In most publications about liberation maneuvers (11–16), therefore 2D pictures are shown of the canal with the clod in different phases of the maneuvers, without any hard supporting evidence, simply because it is not available: only indirect evidence exists based on the observation of nystagmus. These papers are very useful and made to help clinicians to understand the sequence of otolith movements occurring during each step of the maneuvers. But 2D visualization is limited, and therefore we choose to add dynamics to it and to be able to change the projection angle, to see what is assumed to happen as can be seen from different angles at any moment. In this way our simulations will show that changing angulations can affect the outcome of otolith movement, why waiting between steps is required to ensure the clod moves to the starting position and how there may be a failure of a repositioning maneuver. These dynamic simulations can serve as a tool to develop modifications of existing maneuvers and also new maneuvers.

## Methods

The 3D morphology of the inner ear used is as realistic as possible and based on reconstructed MRI images of the temporal bone. DICOM files of MRI images were used to extract the 3D inner ear. The three semi-circular canals and their orientation planes were determined. Angles were taken to quantify the spatial orientation of labyrinthine structures in relation to each other and in relation to aspects of the cranium. Our results agreed with those reported in other studies (3, 5, 17–20). In our simulation model, a thin tube was inserted at the center of each canal. A crystal resembling otoconial debris was put inside these canals while considering the particle drag (friction caused by the fluid on the object immersed in it) and the gravity acting on the crystal. A crystal size of 0.7 mm was taken to represent the otoconia and the diameter of tube of 1.5 mm has been used in the simulations. A fluid linear drag of 35N and fluid angular drag of 0.05N was applied. These parameters helped to study the otoconial movement during the maneuver. The simulation was created on Unity 3D Game engine software. The software allowed us to place the particle in more than one canal at the same time. A humanoid was animated within Autodesk Maya with precise angles for each step of different maneuvers. The head was linked to the semi-circular canals such that when the head moves, the associated canals are stimulated. As the humanoid animates into various positions, the crystal within its inner ear moves because of gravity.

Our goal was to study the effect of gravity on these particles, causing them to move toward the lowest dependent position when the head is moved at different angles while performing the maneuver. Different simulations were developed to understand these alterations in detail. This paper describes the simulation for different variations of the Semont and Yacovino maneuver.

## Maneuvers

Various maneuvers have been described for treatment of different BPPV variants. For PC-BPPV, the maneuvers described by Epley (11) and Semont et al. (12) are most commonly used. The Epley's maneuver uses the effect of gravity on the otolith particle to move it toward the utricle while Semont's maneuver works on the principal of acceleration and gravity (21–24). This paper describes the simulation of Semont's maneuver along with variations for posterior canal. The treatment for AV-BPPV by simulation of Yacovino maneuver and its variations is also described.

### Variations of Semont's Maneuver

Semont's maneuver is used to treat posterior canal Canalolithiasis and Cupulolithiasis (Refer to Table 1). Here, we have considered four variants to understand which one of them can deliver the best results. The first simulation is of the classic Semont's liberatory maneuver for right posterior BPPV. The steps followed are described below:

1. The patient is made to sit on the bed with legs hanging down.
2. The patient's head is turned to the healthy left side by 45°.
3. The patient is then moved to the right side-lying position at an angle of 90° with the head pointing upwards.
4. The patient is now rapidly taken to the opposite side-lying position by swinging the body by 180°.
5. Finally, the patient is brought upright, and after that, the head is turned to the neutral position.

**Table 1:** Types of BPPV and therapeutic maneuvers (PC–BPPV, Posterior canal BPPV; HC–BPPV, Horizontal canal BPPV; AC–BPPV, Anterior canal BPPV; QLR, Quick liberatory rotation); FPP, Forced Prolonged Positioning (11–16, 25).

**Type of BPPV**		**Therapeutic Maneuver**
PC–BPPV	Long arm canalithiasis	Epley/Semont
	Short arm canalithiasis	Brisk epley/Brisk semont/Side lying position with vibrator
	Non-ampullary end canalithiasis	Yacovino/QLR
	Canal side cupulolithiasis	Semont
	Utricular side cupulolithiasis	QLR from opposite side
HC–BPPV	Canalithiasis	Barbecue/Gufoni/FPP
	Cupulolithiasis	Barbecue/Modified gufoni/Zuma/FPP
AC–BPPV	Canalithiasis/Cupulolithiasis	Yacovino

At step 3, the debris can be seen moving away from the ampulla toward the lowest point of the canal due to gravity acting on it. In step 4, the rapid acceleration leads to a centrifugal force that keeps the clot attached to the membranous labyrinth, bringing the clod in the optimal position so that the clot will fall down driven by gravity and due to the deceleration (inertia of mass) also is launched toward the common crus and onwards to the utricle.

So, the simulator proves that the technique is useful for treating this type of BPPV, but it is important to swing the patient rapidly from the right to the left side (Simulation 1).

Simulation 1–Semont's Maneuver (click to view).

If this acceleration is too low during step 3, the particle may fail to move away from the ampulla. Simulation 2 shows that when step 2 is not done with sufficient high acceleration, the clot falls back into the ampullary arm. This simulation emphasizes the need for rapid acceleration in step 4 and how a slow movement can result in failure of the maneuver.

Simulation 2–Semont's Maneuver with slow acceleration (click to view).

Obrist et al. (26) described the Semont's Plus maneuver. In this modification, when the patient is brought to the side-lying position in Step 2, the head angulation is increased from 90 to 120 degrees. This brings the posterior canal to a position where gravity can act more effectively on the particle (Simulation 3). This makes the modified maneuver more efficient than the classic Semont's liberatory maneuver. It was also seen that the maneuver works well-even if the speed of the maneuver is decreased, unlike the previous variant.

Simulation 3–Semont's Plus maneuver (click to view).

The fourth variant shows what happens when the angulation is reduced on bringing the patient to the side-lying position. This is done by placing a pillow under the head (Simulation 4). This decreases the head angulation causing the otoconial particle to fall back into the canal. This emphasizes that correct head angulation is very important for the maneuver to reposition the particle back into the utricle.

Simulation 4–Semont's maneuver with reduced head angulation (click to view).

### Variations of Yacovino Maneuver

Yacovino maneuver is used to treat BPPV involving anterior semicircular canal, or when the debris is present in the common crus of the posterior semicircular canal. The maneuver consists of 4 steps:

1. The patient is asked to sit with the head facing forward.
2. The patient's head is brought to the head hanging position, 30° below the horizontal.
3. The patient's head is brought quickly forward to the “chin to chest” position while still in the supine position.
4. The patient is brought back to the sitting position.

Simulation 5 explains how the maneuver works for anterior canal BPPV at each step.

In Step 2, when the patient is brought to the head hanging position, the otoconial debris begins to move in the direction away from the ampulla.

Step 3–Gravity facilitates the particle to move toward the common crus.

Step 4–Particle falls back into the utricle.

This maneuver is widely accepted, but when we tried the maneuver in the simulator, we found that it has a high chance of canal switch with the particle entering into the posterior canal while treating the anterior canal BPPV (27).

Simulation 5–Yacovino maneuver (click to view).

In a variant of the described Yacovino maneuver, the patient is brought from the head hanging position to the sitting and kept there for 20 s. Finally, the neck is flexed forward after 20 s (Simulation 6). This demonstrates a better way of repositioning the particle back into the utricle than the classic Yacovino maneuver with a lesser chance of particle entering into the posterior canal.

Simulation 6–Yacovino maneuver with head brought straight up (click to view).

In the third simulation, when the patient is brought to the sitting position from the deep head hanging one, the neck is bent immediately (Simulation 7). We can see that due to this, the particle fails to move toward the common crus and instead falls back toward the ampulla.

Simulation 7–Failed Yacovino Maneuver (click to view).

## Results

The model allows a clear visualization of the semicircular canals and movement of otolith debris to the dependent portion of the canal during the maneuvers. The simulation model attempts to show the movement of the particle in a continuous way in three dimensions during the maneuver for better understanding. The whole simulation was created with an aim to understand the dynamics of the otoconial debris with respect to the position of the head.

## Discussion

The simulator is able to change the camera angulation that makes the three-dimensional spatial movement of the head, semicircular canals, and the otoconial debris easier to understand. The user can define the angulation at each step of the maneuver and have a three-dimensional visualization of the canal and the otoconial movement. The simulation developed allows alteration of various parameters like the angulation of the head, the initial position of the otoconial debris in the canal, size and the number of these particles, fluid dynamics of endolymph, and the time of each step of the maneuver. The simulator helps to understand the otoconial movement with respect to the movement of the head. This helps us to understand the optimum plane and angulation required to get the best results. It also helps to understand why the maneuver is ineffective when these planes and angles are not achieved. The mechanism of action of different maneuvers for each type of BPPV could be evaluated. Multi-canal BPPV occurs when there are clots in more than one canal. This can be studied well-using this simulator. Canal switch may be seen during or after BPPV repositioning. Using this simulator, one can understand the different mechanics of the fluid and the canal and thereby can avoid canal switch. In addition, this can also be used as a tool to devise and test the efficacy of new maneuvers.

## Conclusion

A simulator based on the reconstructed human MRI images works as a guidance system during the maneuvers of BPPV. It helps to understand and observe what actually happens when the head moves. It provides a better understanding of what happens on incorrect angulations while performing the maneuver, which can complicate the treatment (e.g., Canal switch) (27). It can be used as a learning and a teaching tool for medical students and practitioners to understand the behavior of the particle present in the canal in relation to head movement. The high-quality 3D visualization of the canal linked to head movement helps to understand the importance of each step of the therapeutic maneuver. It also highlights the important head movements that bring the canals at an angle at which the gravity can act on the particle and remove it from the canal. Correct head angulation is the key to a successful maneuver. Thus, it can provide a thorough explanation for the maneuvers done incorrectly and eliminate the incorrect and unnecessary steps of the maneuver. Multi-canal BPPV is a complicated variant of BPPV as it affects more than one canal of the same or different ears. It is difficult to understand which canal needs to be treated first and what direction the particle moves when one of the affected canals is being treated. For example, if the otoconial debris is present in both the posterior and horizontal canal of the same side, Epley's maneuver will obviously remove the particle from the posterior canal. However, the simulator will also show what happens to the particle present in the horizontal canal due to the maneuver being performed. It can also provide a visual explanation for the need of specific maneuvers for each type of BPPV and why Gufoni maneuver can treat BPPV of the horizontal canal but not the posterior canal BPPV. Due to the recognition of variants of BPPV, more new therapeutic maneuvers have been tried for treatment. The simulator can test and compare the efficacy of these maneuvers.

Currently, the major limitation of this simulator is that it does not entirely represent the population as the orientation of the semicircular canals vary from patient to patient. Our study is based on the orientation obtained from the reconstructed MRI images. We are fully aware that the natural variations in the orientation and morphology have a substantial impact on the validity of the extrapolation to the individual patient. Like various publication describing repositioning maneuvers for BPPV (15, 16, 21, 22), these simulations do not represent the physics of the otoconia. The ideal would be to optimally visualize the otolith movement of each patient, however at present, there are many unknown variables. In this study, the time taken for the particle to move at each step of the maneuver has been accelerated to make it more user-friendly.

However, we experienced that simulators are an effective way to understand all the types of BPPV and their therapeutic maneuvers. Other publications describe the initial and final position of the otolith at each step by two dimensional illustrations. The simulation model attempts to show the movement of the particle in a continuous way in three dimensions for better understanding. This tool provides insights that can lead to a more accurate diagnosis and treatment of BPPV.

## Data Availability Statement

The raw data supporting the conclusions of this article will be made available by the authors, without undue reservation.

## Author Contributions

AB conceptualized the labyrinth simulation in 3D to understand the movement of the otolith clot during head movement in BPPV patients. She has written the manuscript of the publication. HK has given essential inputs to improve the simulation model in various stages of its development, notably in clot movement and visualization, and optimized the text. RB with his technological background and deep understanding of fluid dynamics and labyrinthe disorders. RB has developed the software simulation of BPPV. He has worked on demonstrating the effect of changing head positions and angulations on otolith clot movement in different canalolith repositioning maneuvers in 3 dimensions. All authors contributed to the article and approved the submitted version.

## Conflict of Interest

The authors declare that the research was conducted in the absence of any commercial or financial relationships that could be construed as a potential conflict of interest.
